# Extensive Recombination Due to Heteroduplexes Generates Large Amounts of Artificial Gene Fragments during PCR

**DOI:** 10.1371/journal.pone.0106658

**Published:** 2014-09-11

**Authors:** Jia Liu, Hongshuo Song, Donglai Liu, Tao Zuo, Fengmin Lu, Hui Zhuang, Feng Gao

**Affiliations:** 1 Department of Microbiology, Peking University Health Science Center, Beijing, China; 2 Department of Medicine, Duke University Medical Center, Durham, North Carolina, United State of America; 3 National Engineering Laboratory for AIDS Vaccine, College of Life Science, Jilin University, Changchun, Jilin, China; Centro Nacional de Microbiología - Instituto de Salud Carlos III, Spain

## Abstract

Artificial recombinants can be generated during PCR when more than two genetically distinct templates coexist in a single PCR reaction. These recombinant amplicons can lead to the false interpretation of genetic diversity and incorrect identification of biological phenotypes that do not exist *in vivo*. We investigated how recombination between 2 or 35 genetically distinct HIV-1 genomes was affected by different PCR conditions using the parallel allele-specific sequencing (PASS) assay and the next generation sequencing method. In a standard PCR condition, about 40% of amplicons in a PCR reaction were recombinants. The high recombination frequency could be significantly reduced if the number of amplicons in a PCR reaction was below a threshold of 10^13^–10^14^ using low thermal cycles, fewer input templates, and longer extension time. Heteroduplexes (each DNA strand from a distinct template) were present at a large proportion in the PCR products when more thermal cycles, more templates, and shorter extension time were used. Importantly, the majority of recombinants were identified in heteroduplexes, indicating that the recombinants were mainly generated through heteroduplexes. Since prematurely terminated extension fragments can form heteroduplexes by annealing to different templates during PCR amplification, recombination has a better chance to occur with samples containing different genomes when the number of amplicons accumulate over the threshold. New technologies are warranted to accurately characterize complex quasispecies gene populations.

## Introduction

PCR has played a vital role in quickly obtaining gene fragments from a variety of biological samples [1]. However, when two or more genetically related but divergent genomes were present in the samples, artificial recombinant amplicons between different templates are frequently generated during PCR [2]–[9]. PCR-mediated recombinants can significantly alter the genes or gene fragments through exchanging large parts of sequences between different genomes. These artificial recombinants can contribute to the false interpretation of genetic diversity in sample as well as incorrect identification of novel gene species and new biological phenotypes that do not exist *in vivo*.

PCR-mediated recombination was recognized soon after PCR was widely used [2]. Previous studies showed that the thermal cycles, templates inputs, extension time and enzymes could affect the recombination frequencies [2], [5], [10]–[13], but the precise frequency and mechanisms of recombination have not been well studied due to the limitations of previous methodologies. Analysis of sequences obtained by cloning individual PCR amplicons [3], [5] or single genome sequencing (SGS) is commonly used, but these methods are labor intensive and limited by the number of available sequences [7], [11], [12]. Restriction fragment-length polymorphism [10], [14], probe hybridization [2], [3], [15] and phenotype rescue screening [16], [17] can detect limited genetic markers. However, the sensitivity and accuracy of these methods are not ideal for detection of recombinants. The next generation sequencing (NGS) methods can analyze thousands of sequence reads, but are limited by the sequence length and the requirement for the large number of templates [4], [6], [12], [13], [18]. Recently, the complex microbe quasispecies population in each individual infected by HIV or HBV [12], [19]–[21] and the immunoglobulin repertoire in single individuals [22]–[26] have been studied by NGS. Each read generated by NGS is often independently analyzed and functionally characterized to study the low frequency viral genomes or immunoglobulin molecules in the quasispecies population. A recent study showed that the recombination frequency for less abundant species in a quasispecies population could exceed 70% by NGS analysis [6]. Thus, it will be critical to understand how NGS sequences are affected by recombination during the bulk PCR amplification step.

Heteroduplexes generated by annealing an incompletely extended primer to a heterologous template [2], [12], [14], [27] was considered as the main cause for generation of recombinants during PCR. However, none of existing methods can directly detect recombinant events in heteroduplex templates as individual double-strand DNA molecules. Therefore, the mechanisms for recombination during PCR remain unresolved. We have recently developed a parallel allele-specific sequencing (PASS) assay [28], which can detect rare genomes as low as 0.01% among thousands of DNA molecules in a single assay [29], [30]. The advantage of the PASS assay is direct analysis of each DNA molecule, determination of recombinant genomes through linkage analysis of multiple sites in each individual genome, and characterization of both strands of the DNA molecules at the same time [28], [30]–[32]. In this study, we used the PASS method to determine how the numbers of templates, thermal cycles and extension time affected the recombination frequency and whether heteroduplex DNA molecules could result in higher recombination frequencies.

## Materials and Methods

### PCR templates and conditions

Two plasmids containing full-length HIV-1 subtype B strains NL4-3 and 89.6 were used as DNA templates. To investigate the influence of the sequence homology on recombination frequency, two genetically more similar plasmids (1B7 and 1D1), which were genetic variants of the HIV-1 WEAU strain, were also studied. A partial *pol* gene fragment (1307 bp) was amplified with primers BGF2 (5′-ACAACAACTCCCCCTCAGAAGCAGGAG-3′ nt2194-2220 in HXB2) and RT3In (5′-CACTCCATGTACCGGTTCTTTTAG-3′ nt3477–3500). All plasmids were linearized with restriction enzyme NotI before PCR. The PCR amplification was carried out in a 50 µl reaction mix consisting of 1.25 units Platinum Taq DNA Polymerase High Fidelity (Invitrogen Corp., Carlsbad, CA), 0.2 mM each deoxynucleoside triphosphate (dNTP), 0.2 µM of each primer, 2 mM MgSO_2_, equal copy numbers of each template (from 10^1^ copies to 10^7^ copies) and the buffer supplied by the manufacture. The standard thermal cycling conditions were as following: 1 cycle of 94°C for 5 min; 30 cycles of denaturation at 94°C for 30 sec, annealing at 50°C for 45 sec, and extension at 72°C for 2 min; and a final extension step at 72°C for 10 min.

### Detect PCR recombination by PASS

The PCR amplicons were directly subject to PASS assay to determine the recombination frequency as previously described [28], [30]–[32]. In order to obtain well isolated immobilized PCR amplicons (polonies) in the PASS gel to precisely define recombinant amplicons, PCR products were diluted to a concentration that would yield ∼400–600 polonies per gel. Briefly, 20 µl of 6% acrylamide gel mix, containing 1 µM acrydite-modified reverse primer PAR2a-5 (5′Acr-AATCCCTGCATAAATCTGACTTGCCCAAT-3′ nt3343–3371), diluted PCR products, 0.3% diallyltartramide, 5% Rhinohide, 0.1% ammonium persulfate (APS), 0.1% N,N,N′,N′-tetramethylethylenediamine (TEMED) and 0.2% bovine serum albumin (BSA), was used to cast the gel on a bind-saline (Amersham Biosciences, Piscataway, NJ) treated glass slide. The in-gel PCR amplification was then performed in a PTC-200 Thermal Cycler with a mix of 1 µM forward primer SP3 (5′-ATAATTGGAAGAAATCTGTTGACTCAGATTGG-3′ nt2502–2533), 0.1% Tween-20, 0.2% BSA, 1xPCR buffer, 0.1 mM dNTP mix, 3.3 units of Jumpstart Taq DNA polymerase (Sigma, St. Louis, MO), and H_2_O (up to 300 µl) under a sealed SecurSeal chamber (Grace Bio-Labs, Inc., Bend, OR). The amplicon size was 870 bp. The PCR conditions were 94°C for 3 min; 65 cycles of 94°C for 30 sec, 56°C for 45 sec, and 72°C for 3 min; and 72°C for 6 min. After amplification, the PCR products amplified from a single molecule accumulated around the original template and formed a distinct polony. After in-gel PCR, the gels were treated with denaturation solution to remove the free DNA strands. Single base extension (SBE) was then performed with two different bases specific for those in each parental plasmid using sequencing primers that annealed just upstream of the target sites. The polonies in each gel were then sequentially interrogated by six SBE reactions with different primers. After each SBE, the gel was scanned to acquire images with a GenePix 4000B Microarray Scanner (Molecular Devices, Sunnyvale, CA).

### PASS data analysis

The two channel images (Cy5 for the bases from one template and Cy3 for the bases from the other template) were first cropped with Picture Window Pro3.5 (Digital Light & Color, Belmont, MA) to remove the edge area containing no specific signals. The cropped images were then analyzed with the Progenesis PG200 software (Nonlinear Dynamics, Durham, NC). After background subtraction, normalization, and spot filter setting, only unambiguous spots at both channels were included for further analysis. The normalized pixel count data at two mutation sites at each spot were exported into an Excel file with a unique identifier. By comparing each spot's normalized values at both channels, the different viruses were identified based on the base identity, and the percentage of each compared genome in the population was then determined. Then all spots in six gels were examined manually to identify homoduplexes (identical bases in both DNA strands) and heteroduplexs (bases in two DNA strands from different templates) generated during PCR. The linkage pattern of six bases in each amplicon was determined using the Linksys program as previous described [28], [30]–[32].

### Next generation sequencing (NGS) analysis

A total of 35 different HIV-1 whole genome plasmid clones were mixed together (1 ng/µl each). The mixture was then diluted to the final concentrations of 3.5×10^4^, 3.5×10^5^ and 3.5×10^6^ copies/µl (10^3^,10^4^ and 10^5^ copies/µl for each clone, respectively). One microliter of each dilution was amplified by 20, 25, 30 or 35 PCR cycles using the forward primer (5′-*TCGTCGGCAGCGTCAGATGTGTATAAGAGACA*GGTAGCAAAAGAAATAGTAGCTAGCTGTGATAA-3′; nt 4323-4354) and the reverse primer (5′- *GTCTCGTGGGCTCGGAGATGTGTATAAGAGACA*GATGAATACTGCCATTTGTACTGCTGT-3′; nt4749–4775). The plain letters are HIV-1 specific while the italic letters are complementary to the index primers from the Illumina Nextra Index Kit (Illumina, San Diego, CA). The first round PCR products were purified using the MinElute PCR purification kit (Qiagen, Valencia, CA) and subjected to an additional 10 cycles of PCR using the index primers provided by the Illumina Nextra Index Kit (Illumina, San Diego, CA) to add the unique indexes and adaptors to both ends of the amplicons. The second round PCR products were purified to eliminate the primer dimer, quantified by qPCR on an ABI 7300 realtime PCR machine, and sequenced using a two-direction 600 cycle reagent kit on MiSeq (Illumina, San Diego, CA). Each pair of fastq files containing the sequences from read 1 and read 2 were merged by FLASH [33]. The merged fastq files were filtered by Galaxy [34], using the parameter of ≤3 bases with lower than of Q score 30 in each read. The filtered reads were then aligned to the reference sequence using the BWA [35]. The frequencies of all 35 viral sequence and their recombinants were determined by detecting the haplotypes for 139 informative sites that were specific for each virus using Nautilus [36].

## Results

### Increased recombination frequency during PCR with higher numbers of thermal cycles and templates

**Figure 1:**
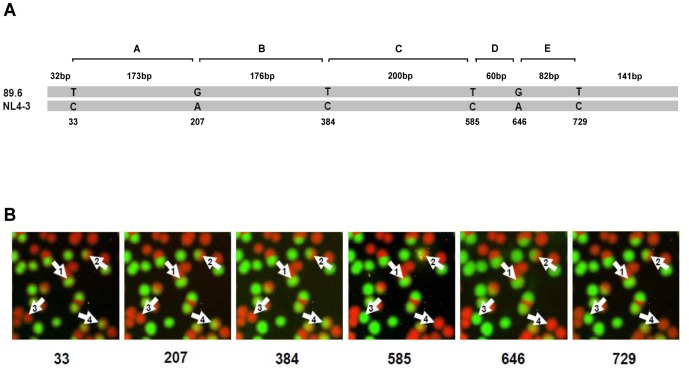
Detection of PCR-mediated recombinants by PASS. (A) Nucleotides used for linkage analysis to identify recombinants are indicated. A partial *pol* gene (870 bp) was amplified. Nucleotides that are distinct at six positions between 89.6 and NL4-3 are shown. The regions between two neighbor nucleotides are named as A through E and the genetic distances between them are shown. (B) Linkage analysis of nucleotides at six positions by PASS. The polonies in the same PASS gel were probed by six sequential SBEs to identify recombinants. Each image represents the results from one SBE. The sequencing primers were named according to the base positions and are indicated at the bottom of the image. Each spot represents an amplicon from a single DNA molecule. The bases in 89.6 were detected by SBE with Cy5-labeled nucleotides (red) and the bases in NL4-3 were detected by SBE with Cy3-labeled nucleotides (green). The numbered arrows indicate linkage analysis results from different double-stranded DNA molecules: (1), homoduplex without recombination; (2), heteroduplex without recombination; (3), homoduplex with a recombination breakpoint between nt384 and nt585; (4), heteroduplex with a recombination breakpoint between nt384 and nt585.

**Figure 2:**
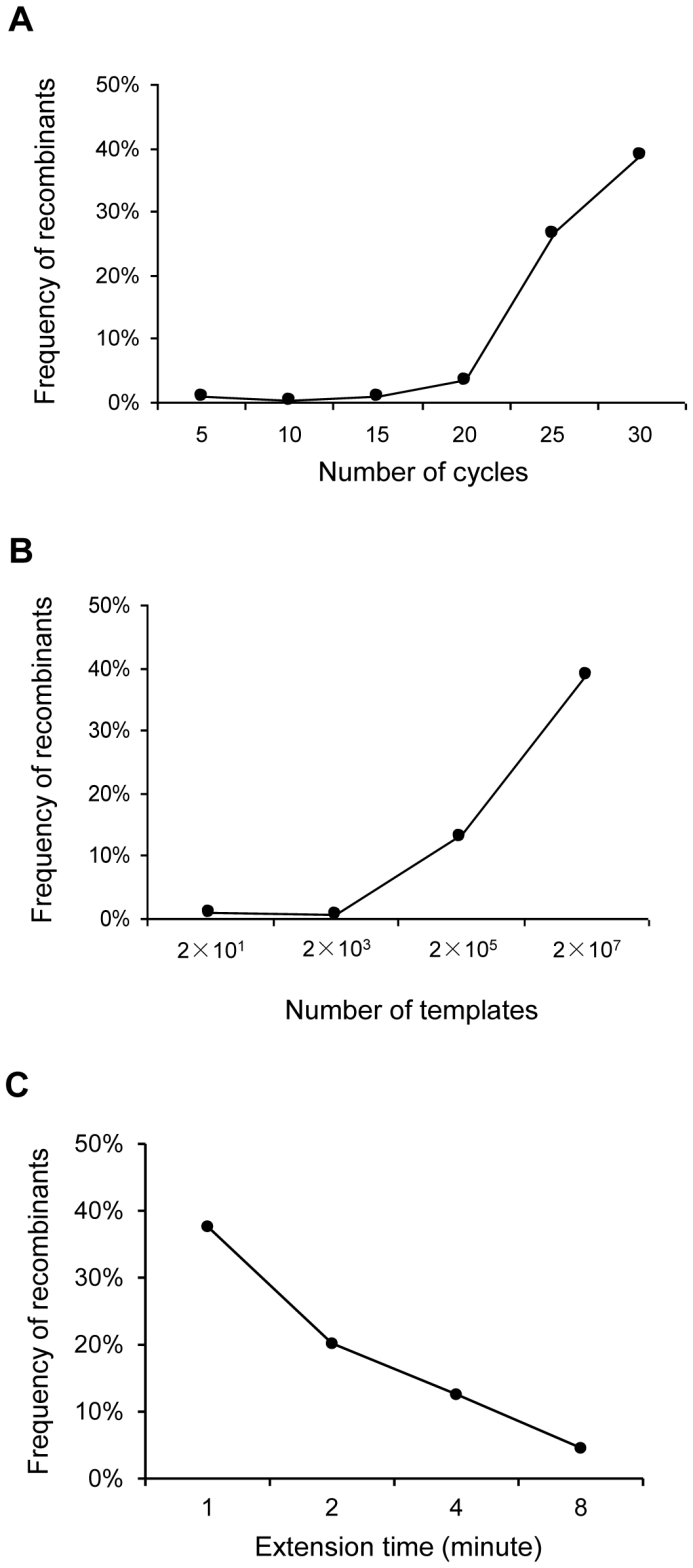
Recombination frequencies at different conditions during PCR. (A) Recombination frequencies were determined at different thermal cycles. Equal amount of NL4-3 and 89.6 plasmids (10^7^ copies per template) were mixed together and co-amplified. The PCR was carried with 5, 10, 15, 20, 25 or 30 thermal cycles. (B) Recombination frequencies were determined with different numbers of templates. Equal amount of NL4-3 and 89.6 plasmids (10^1^, 10^3^, 10^5^ or 10^7^ copies each) was mixed together and co-amplified by 30 cycles of PCR. (C) Recombination frequencies were determined with different extension time. Equal amount of NL4-3 and 89.6 plasmids (10^7^ copies per template) were mixed together and co-amplified. The PCR was carried with different extension time (1, 2, 4 or 8 minutes). The PCR products were analyzed by the PASS assay and the recombination frequency at each condition was determined by linkage analysis of six bases.

To investigate how the number of PCR cycles could affect PCR recombination frequency, equal amounts of NL4-3 and 89.6 plasmids (10^7^ copies each) were mixed together and subjected to PCR amplification at 5, 10, 15, 20, 25 or 30 cycles. The genetic diversity between NL4-3 and 89.6 was 3.1% in the amplified fragment (870 bp). To detect the recombination events generated during PCR, we analyzed six template-specific sites that scattered throughout the amplified gene fragment (Figures 1A; Figure S1A in File S1). An average of 532 (420–697) viral genomes for each thermal cycle condition were analyzed (Table S1 in File S1). The nucleotide identities at six positions in each individual sequence were determined by sequential SBE reactions (Figure 1B). We then performed linkage analysis of all detected six bases on each individual genome to determine the proportions of the parental and recombinant genomes through examination of the linkage patterns as we previously reported [28], [30]–[32]. The recombinants were defined as the sequences that contained bases from both templates. The recombination frequencies were low (0.5%–1%) at cycles 5–15 and they were not significantly different from each other (Chi square test, p>0.3). The recombination frequency increased to 3.6% at cycle 20, which was significantly higher than those at cycles 5–15 (Chi square test, p = 0.002). It continued to increase to 26.7% at cycle 25. At cycle 30, 41.7% of the amplicons were recombinants (Figure 2A; Table S1 in File S1).

We then sought to investigate how the number of templates affected the recombination frequencies during PCR. Different template concentrations (10^1^, 10^3^, 10^5^, and 10^7^ copies for NL4-3 and 89.6 each) were subjected to 30 cycles of PCR amplification and PASS analysis. An average of 529 (487–592) genomes for each template concentration were analyzed (Table S2 in File S1). Only low levels of recombinants (≤1%) were detected at concentrations of 2×10^1^ or 2×10^3^ template copies (Figure 2B; Table S2 in File S1). The recombination frequency significantly increased to 12.9% and 41.7% at concentration of 2×10^5^ and 2×10^7^ template copies, respectively (Chi square test, p<0.001). These results showed that recombinants significantly increased in the PCR reaction when the thermal cycle numbers were ≥25 with 2×10^7^ templates and the templates were ≥2×10^5^ at cycle 30.

### Decreased recombination frequency during PCR with longer extension time

Previous studies showed that the recombination frequency could be reduced when the extension time was increased during PCR [2], [14], [17]. To more precisely understand how the extension time affect recombination, we determine the recombination PCR-mediated frequencies between NL4-3 and 89.6 templates with different extension time by analyzing a large number of amplicons using the PASS assay. Since the recombination frequency was high and varied little within 40–60 seconds with template sizes 500 bp or less [14], [17], no variation in recombination frequency was expected with an amplicon size of 870 bp in our system within 60 seconds of extension. Thus, the extension of 1, 2, 4 or 8 minutes was analyzed with 10^7^ copies of each template. An average of 398 (348–438) genomes for each extension time were analyzed (Table S3 in File S1).

The recombination frequency was highest (37.6%) with the 1-minute extension time (Figure 2C; Table S3 in File S1). It continuously decreased as the extension time increased. The recombination frequency was lowest (4.6%) with the 8-minute extension time, a ∼8-fold reduction from the frequency with the 1-minute extension time. These results confirmed that longer extension time could significantly reduce recombination frequencies.

### Recombination pattern

**Figure 3:**
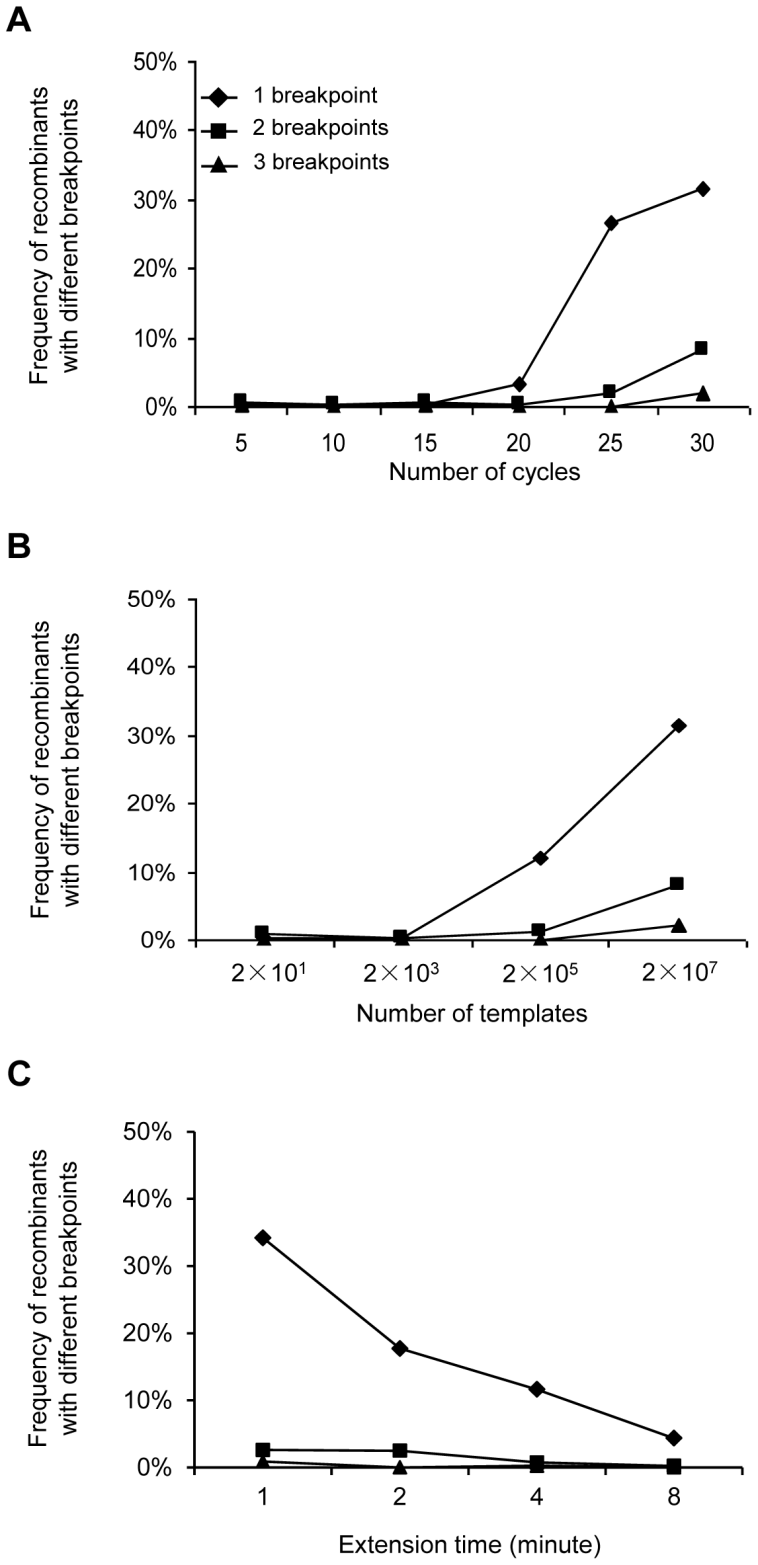
Frequency of recombinants with different recombination breakpoints. Frequencies of recombinants with different breakpoints were determined for PCR with different thermal cycles (A), different template concentrations (B), and different extension time (C). Recombinants with one (diamond), two (square) or three (triangle) breakpoints were determined. No amplicons contained more than three recombination breakpoints.

We next determined the recombination patterns by examining the linkages of all six sites in each amplified genome. All recombination patterns were classified by the location and number of breakpoints (Tables S1, S2 and S3 in File S1). Recombination patterns generated with different template numbers, thermal cycles or extension time were similar. The PCR amplicons with one recombination breakpoint were predominant, accounting for 26.7% and 31.6% of the amplicons at cycles 25 and 30 (2×10^7^ templates), respectively (Figure 3A). The PCR amplicons with two recombination breakpoints were significantly less (Chi square test, p<0.01), accounting for 1.8% and 8.1% of the amplicons at cycles 25 and 30, respectively. The PCR amplicons with three recombination breakpoints were rarely detected within 30 cycles and only 2.0% at cycle 30. When different numbers of templates or extension time were tested, similar results were observed: the PCR amplicons with one recombination breakpoint were predominant while those with two or three breakpoints were less frequent (Figures 3B and 3C).

While the majority of the recombination patterns were present in less than 2% of the amplicons, some recombinants accounted for 4–5% of the total PCR amplicons (Tables S1, S2 and S3 in File S1). No recombinants with more than three breakpoints were detected within 870 bp of the amplified fragment. Overall, rare and random recombination events detected within 20 cycles or with less than 2×10^3^ templates (Figure 3; Tables S1 and S2 in File S1) suggested that the recombination mainly occurred when the total numbers of amplicon templates accumulated to a high level during PCR amplification.

One critical question was whether all those distinct recombination patterns represented real recombination events, not artifacts due to point mutations generated during PCR. Among 40 detected recombination patterns, 11 patterns contained bases from different templates at only one of the six sites (Tables S1, S2 and S3 in File S1). To determine whether the recombinants with only a single base from the other template were due to the PCR error, we performed PCR with the NL4-3 or 89.7 template alone (10^7^ each) for 30 cycles and determined the frequencies of mutations that matched the base in the counterpart template at all six sites by PASS. The mutation rates were generally similar at all six sites in both viruses although it was slightly higher (>10 mutations per site) at three positions (Table 1). Overall the mutation rate was 0.08%, similar to previously reported PCR error rate [37], [38]. In addition, no linked mutations were detected in any PCR amplicons, indicating that PCR alone did not generate the recombinants. Since the recombination frequencies (∼1%) at low thermal cylce and template conditions were more than 10 fold higher than the background mutation rate (0.08%) and since more than half of the recombinants contained at least two bases unique to the counterpart template, the PCR error rate should not significantly affected the analysis of recombinant events generated during PCR.

**Table 1:** PCR error rate with individual template.

Template	No. of genomes	No. of mutations at each site						Total	PCR error rate per site (%)
		33	207	384	585	646	729		
89.6	7671	13	3	6	6	4	14	46	0.1
NL4-3	6359	5	10	2	3	5	0	25	0.07
Total	14030	18	13	8	9	9	14	71	0.08

### Higher PCR recombination frequency between templates with greater similarity

**Figure 4:**
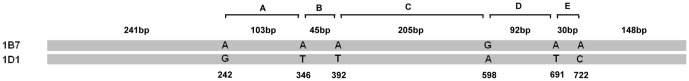
Recombination analysis of two low genetic diversity templates during PCR. A partial *pol* gene (870 bp) was amplified from two genetic variants (1B7 and 1D1) of WEAU. Nucleotides that are distinct at six positions in 1B7 and 1D1 are shown. The regions between two neighbor nucleotides are named as A through E and the genetic distances between them are indicated.

PCR recombination frequency was further studied with templates with higher similarity. WEAU 1B7 and 1D1 plasmids were two variants derived from WEAU partial *pol* gene (Figure S1B in File S1). The genetic difference between them was 0.8%, which was about four fold lower than that between 89.6 and NL4-3 (Table 2). Two templates (1000 copies each) were mixed together and subjected to 30 cycles of PCR amplification. The same 870 bp PCR fragment was analyzed by PASS. There were seven base differences between 1B7 and 1D1 in this region, but two of them were next to each other (Figure S1B in File S1). Thus, six sites were analyzed to determine the recombination events generated during PCR (Figure 4). The recombination frequency was 3.1% between genetically more similar 1B7 and 1D1 templates (Table 2). Under the same condition, the recombination frequency was 0.8% between genetically more divergent NL4-3 and 89.6 templates. These results demonstrated that the four-time lower genetic diversity between templates increased the recombination frequency by four folds during PCR.

**Table 2:** Comparison of recombination frequencies between templates with high and low genetic diversities.

Template	Diversity (%)	No. of genome analyzed	No. of recombinants	% of recombinants
89.6/NL4-3	3.1	519	4	0.8
1B7/1D1	0.8	390	12	3.1

Note: 30 cycles of PCR with 1000 copies of each template.

We next analyzed the recombination frequency between each sites in the templates. The recombination frequencies were significantly higher between the sites that were separated by more than 170 bp than those by less than 100 bp (Chi square test, p<0.01) between more divergent 89.6 and NL4-3 templates (Table 3). We also observed the similar trend for the less divergent 1B7 and 1D1 pair. However, the differences were not significant (p>0.1).

**Table 3:** Recombination frequency between sites at different length.

NL4-3/89.6			1B7/1D1		
Region	Length (bp)	Frequency of recombinants (%)	Region	Length (bp)	Frequency of recombinants (%)
C	200	4.07	C	205	6.68
B	176	4.37	A	103	5.83
A	173	5.52	D	92	5.1
E	82	1.43	B	45	4.74
D	60	1.73	E	30	3.16

### Higher recombination frequency in heteroduplexes than in homoduplexes

To minimize the possibility that two templates were placed together, we diluted the PCR products to the concentration at which the amplified products from each individual DNA molecule were well separated from each other. However, a large proportion of individual PCR amplicons contained bases from both parental templates (Figure 1B) at the template concentrations at which only rare such polonies could be observed when only one template was analyzed as in our previous studies (Figure S2 in File S1) [28]–[32]. These results demonstrated that those were individual PCR amplicons consisting of DNA strands from both parental templates. Thus, all PCR amplicons could be classified into two forms: homoduplex that contained both DNA strands from the same parental template and heteroduplex that contained the DNA strands from different parental templates.

**Figure 5:**
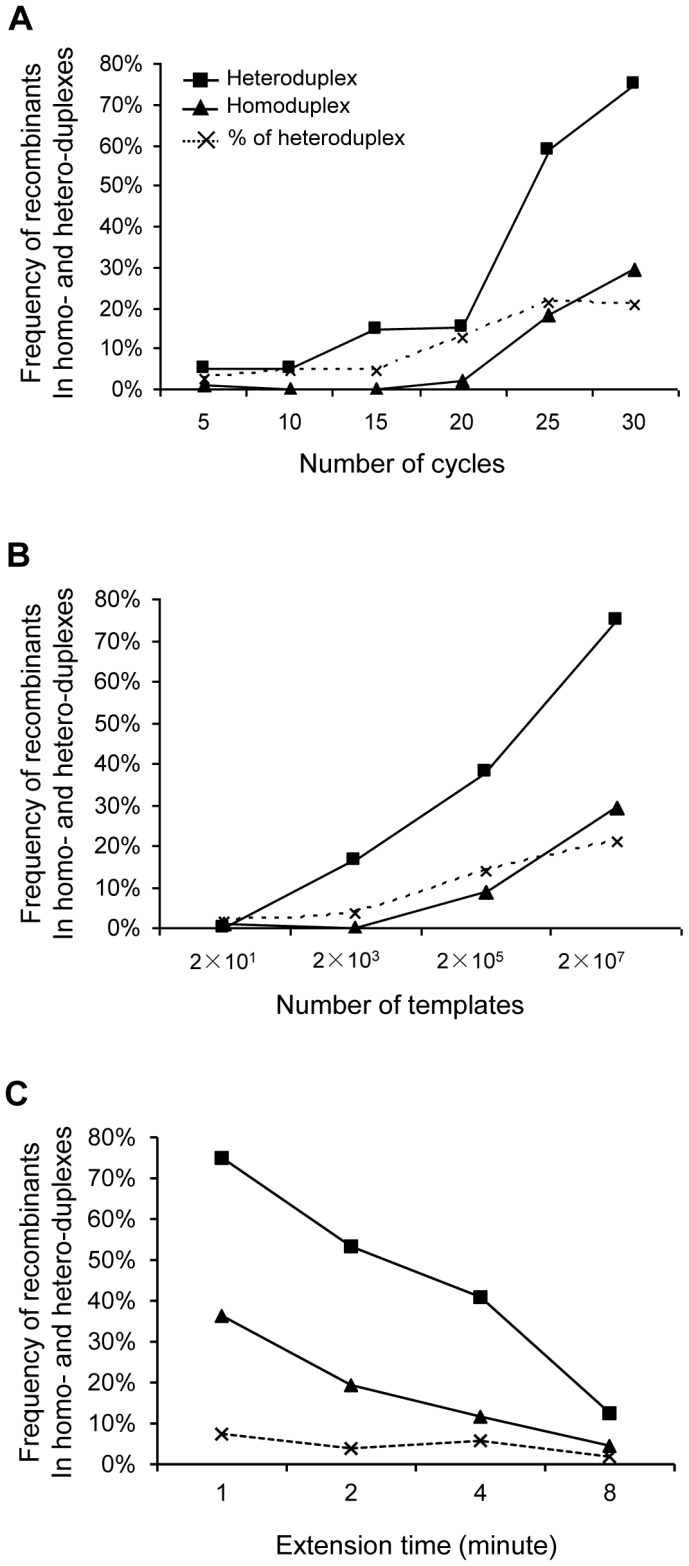
Recombination frequency in heteroduplexes and homoduplexes generated during PCR. Heteroduplex (two DNA strands from different parental templates) and homoduplex (both DNA strands from the same parental template) were determined by PASS. The frequencies of recombinants in heteroduplex (square) and homoduplex (triangle) were determined by linkage analysis. The frequencies of heteroduplexes at different thermal cycles (A), different template concentrations (B) and different extension time (C) are indicated by dotted line.

The heteroduplexes were present at low levels (2.7%–4.6%) within 15 cycles when 2×10^7^ templates were used (Figure 5A). It significantly increased to 12.7% at cycle 20 and continued to increase to 21.3% and 20.9% at cycles 25 and 30, respectively. The heteroduplexes were few with 20 copies of NL4-3 and 89.6 templates after 30 PCR cycles, but it then significantly increased to 3.5%, 13.7% and 20.9% when the copies of the templates were increased to 2×10^3^, 2×10^5^ and 2×10^7^, respectively (Figure 5B). Importantly, as the number of the heteroduplexes increased during PCR, the percentages of the heteroduplexes containing the recombinant DNA strands also went up. The frequency of recombinants in the heteroduplexes was about 5% at cycles 5 and 10, increased to about 15% at cycles 15 and 20, but reached 58.5% and 75% at cycles 25 and 30, respectively (Figure 5A). Similarly, recombinants were not detected with 2×10^1^ copies of templates, but the frequency of recombinants in the heteroduplexes quickly increased to 16.7%, 38.0% and 75% with 2×10^3^, 2×10^5^ and 2×10^7^ copies of templates, respectively (Figure 5B). Much lower frequencies of recombinants were found in homoduplexes than in heteroduplexes under the same condition (Figure 5). For example, heteroduplexes accounted for 20.9% of the PCR amplicons after 30 cycles with 2×10^7^ templates, but recombinants were found in 75% of the heterodulexes. In contrast, the recombinants were only found in 29.5% of homoduplexes which accounted for 79.1% of the PCR amplicons. Similar results were also observed for PCR products generated with various extension time (Figure 5C).

To investigate how frequent heteroduplexes formed, we performed one cycle PCR and simple hybridization with NL4-3 and 89.6 templates. Since no gene amplification occurred, high copies of NL4-3 and 89.6 templates (10^11^ copies for each) were mixed together for detection of heteroduplexes. After one cycle primer extension (one round of denaturation, annealing and elongation with primers) or hybridization (one round of denaturation and annealing without primers), the products were subjected to PASS assay. The frequencies of heteroduplexes were similar in both conditions (16.5% and 18.8% for single round PCR and hybridization, respectively) (Table 4). However, recombinants were only detected in the single round PCR products, and more recombinants were found in heteroduplexes than in homoduplexes. These results demonstrated that the partially synthesized nascent DNA strands could disassociate with the one template and realigned to the counterpart template during single round elongation step and generated recombinant DNA fragments after continuous elongation using the counterpart template.

**Table 4:** Frequency of recombinants in heteroduplexes and homoduplexes.

	Homoduplex			Heteroduplex		
	No. of molecules	% of homoduplexes	No. of recombinants	No. of molecules	% of heteroduplexes	No. of recombinants
One cycle PCR	475	83.5	2	94	16.5	4
Hybridization	329	81.2	0	76	18.8	0

### High frequency recombination during simultaneous amplification of multiple distinct templates

To investigate how multiple distinct templates in the sample could affect the recombination frequency, we generated a mixture of 35 different HIV-1 genomes. One pair of highly conserved primers among all 35 viral genome was designed to amplify a partial *pol* gene (452 bp) from these HIV-1 genomes. A total of 139 unique sites among those 35 HIV-1 genomes were required to distinguish each virus from others. Since such a large number of sites could not be analyzed by the PASS assay, the recombination frequency was determined by analyzing a large number sequences using the NGS method. To fully determine the impact of multiple templates on the recombination frequency during PCR, three concentrations of templates (3.5×10^4^, 3.5×10^5^ and 3.5×10^6^ copies/µl) and four total thermal cycle numbers (30, 35, 40 and 45) were performed. Positive PCR reactions were obtained for all conditions, except with 3.5×10^4^ template copies during the initial 20-cycle amplification due to the low numbers of templates and thermal cycles. The first round PCR products (at 20, 25, 30 or 35 cycles) were then subjected to an additional 10 cycles of PCR to add the unique indexes and adaptors to both ends of the amplicons for NGS. The individual PCR amplicons were analyzed by sequencing 300 bp from each direction. Both reads from each amplicon were stitched together through the shared overlap region to generate a final sequence (∼452 bp) for each amplicon. The frequencies of sequences that identical to or different (recombinant) from the parental sequences were determined by analyzing the linkage patterns of 139 unique bases in each sequence using Nautilus [36]. An average of 325,719 (205,152–406,988) raw reads and 198,022 (110,412–262,456) final sequences that could be successfully aligned to HIV-1 reference sequence were obtained for each PCR condition (Table 5).

**Table 5:** Determination of recombinant frequencies in PCR with 35 distinct templates by next generation sequencing.

Number of cycles	Copy of templates	Total raw reads	Merged reads	Filtered sequences	Number of sequences aligned to reference	Recombinant sequences	
						Number	Percentage
30	3.5×10^5^	291,206	288,721	200,665	188,891	24,833	13%
	3.5×10^6^	406,988	403,870	277,553	262,456	40,078	15%
35	3.5×10^4^	205,152	203,512	117,362	110,412	13,812	13%
	3.5×10^5^	383,225	380,152	245,514	232,128	39,066	17%
	3.5×10^6^	349,839	347,025	226,771	214,915	75,407	35%
40	3.5×10^4^	257,073	254,828	166,013	154,172	24,496	16%
	3.5×10^5^	353,325	350,222	228,543	214,317	52,324	24%
	3.5×10^6^	300,406	298,262	204,089	192,880	85,584	44%
45	3.5×10^4^	310,392	307,886	203,912	191,644	44,856	23%
	3.5×10^5^	381,909	378,724	245,945	231,893	107,881	47%
	3.5×10^6^	343,394	340,447	194,522	184,538	112,661	61%

**Figure 6:**
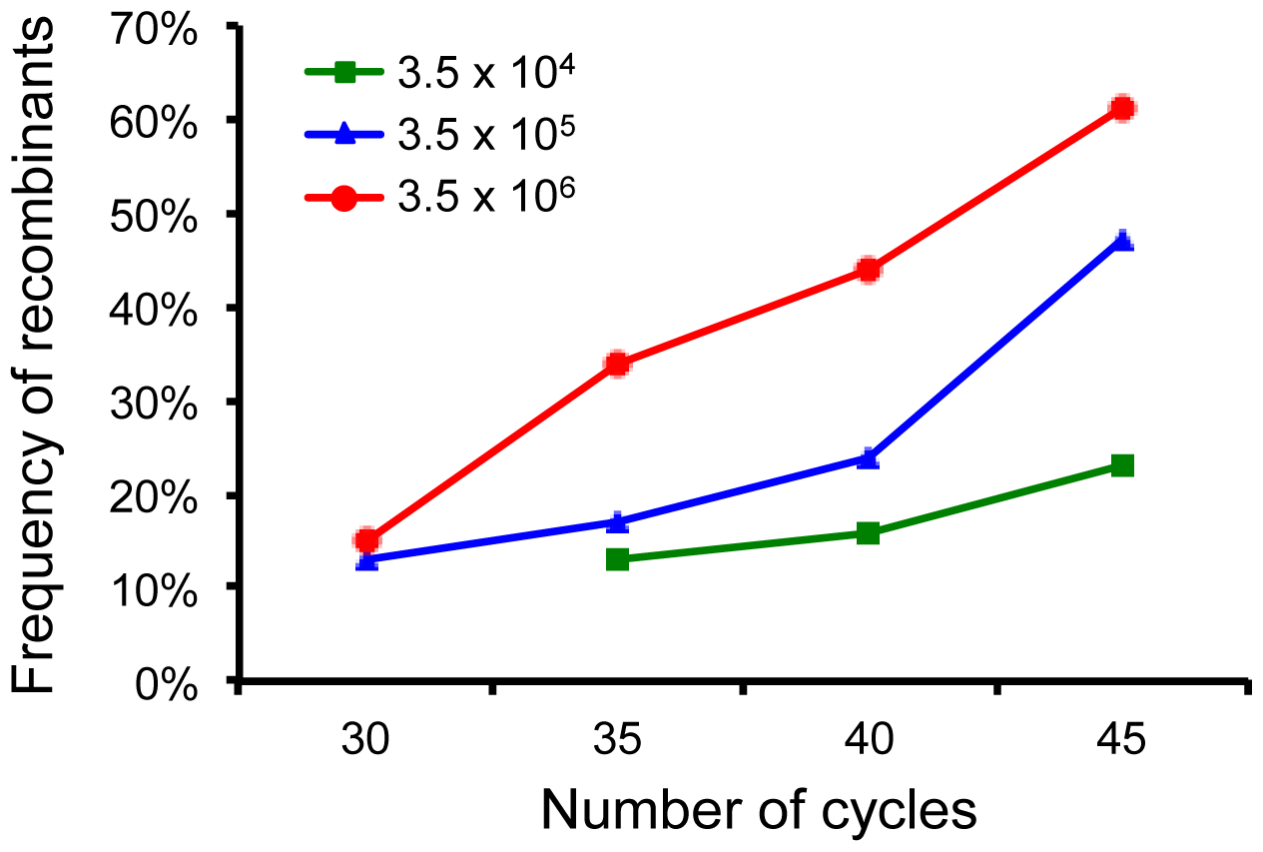
Recombination frequency during simultaneous amplification of multiple distinct HIV-1 genomes by next generation sequencing. A mixture of 35 genetically distinct HIV-1 genomes was subjected to PCR amplification. The PCR was performed with different copies of templates (3.5×10^4^, 3.5×10^5^ or 3.5×10^6^ copies) using different thermal cycle numbers (30, 35, 40 and 45). The PCR products were sequenced using a two-direction 600 cycle reagent kit on MiSeq. The merged sequences from two overlapping reads of the same cluster were then aligned to the HIV-1 reference sequence. The frequencies of all 35 parental sequence and their recombinants were determined by linkage analysis of 139 informative sites in each amplicon sequence using Nautilus [36].

At low thermal cycles and low template numbers (30 cycles with 3.5×10^5^ copies, 30 cycles with 3.5×10^6^ copies, or 35 cycles with 3.5×10^4^ copies), the recombination frequencies were relatively low (13% or 15%) (Figure 6 and Table 5). This was similar to what previously reported with two templates in the PCR reaction [12]. Recombination frequencies became higher when the number of thermal cycles or the template numbers increased. When the PCR was carried out with 3.5×10^6^ copies of templates after 45 cycles, as high as 61% of the amplicons were recombinants (Figure 6). These results showed that multiple genetically distinct templates in the same PCR reaction could lead to high recombination frequencies.

## Discussion

PCR is a powerful tool to study low copy genomes as well as quasispecies genetic variants in a variety samples [1], [11], [12], [19]–[26], [39]–[41]. However, when multiple different genomic variants in a sample were amplified together, the recombinants generated during PCR can lead to the false interpretation of genetic diversity in the sample, incorrect identification of novel gene species, and new biological phenotypes that do not exist *in vivo*. To avoid such artificial recombinants, SGS techniques were developed to obtain sequences free of recombination from a quasispecies population by amplification of individual genomic templates [11], [39], [42]–[44]. However, the quasispecies genomes of human pathogens and immunoglobulin repertoires were recently characterized by NGS, which requires a bulk PCR amplification of highly complex quasispecies populations [6], [19]–[26]. Therefore, understanding how the recombination frequency is affected by PCR conditions will help to minimize the PCR-mediated recombinants in bulk PCR amplification. Previous studies have showed that the thermal cycles, templates inputs, extension time and enzymes could impact generation of recombinants during PCR [2], [5], [10]–[13]. However, how exactly those factors affect recombination have not fully understood since a large number of relatively long sequences from individual amplicons were not available from various PCR conditions for analysis. In this study, we demonstrated that the higher numbers of thermal cycles and templates could significantly increase the proportions of artificial recombinants in the PCR products. In a standard PCR condition (2×10^7^ templates and 30 cycles), 41.7% of the PCR products were recombinants within an 870 bp gene fragment. Such a higher level of artificial recombinants can significantly affect accurate analysis of a quasispecies genome population obtained by the bulk PCR amplification. However, the longer extension time can significantly reduced recombination frequencies. Thus, when it is not possible to characterize a quasispecies genomic population by SGS, it is important to use minimum numbers of templates and thermal cycles as well as longer extension time to minimize the PCR-mediated recombination.

By directly characterizing PCR amplicons using the PASS assay that can simultaneously analyze thousands of genomes and determine the linkage of bases at multiple sites in each individual genome, we found that heteroduplexes in the PCR amplicon population continuously increased (up to 21%), and the recombination frequency were significantly higher in heteroduplexes (75%) than homoduplexes (29.5%). Thus, our results demonstrated that disassociation of the incompletely extended primer from one template and annealing to a different template was the main mechanism for frequent recombination during PCR. The heteroduplex as the cause for generation PCR-mediated recombinants was previously hypothesized but not proven since all previous methods could not directly analyze heteroduplexes [2], [12], [14], [27]. When the numbers of amplicons were low in the PCR reaction, the chance for heteroduplexes to form was small and recombination occurred rarely. However, when the amplicons accumulated over the threshold of 10^13^ –10^14^ (estimated based on 20 cycles with 2×10^7^ templates or 30 cycles with 2×10^3^ templates), the prematurely terminated primer extension fragments could have a better chance to misalign to different templates and form heteroduplexes that would result in recombinant amplicons.

Recombinants present in individual heteroduplexes would not be detected by other sequencing methods in which the individual double-strand DNA heteroduplexes will be subjected to additional PCR amplification in solution or cloned into plasmids before determination of amplicon sequences. In either case, each strand in the heteroduplex will be separated. In contrast, in the PASS assay, the individual double-strand DNA molecules will be amplified together in a semi-solid acrylamide gel and both DNA strands in the heteroduplexes can be simultaneously analyzed. This unique feature of the PASS assay will also allow it to determine heterozygous alleles present in the same double-strand DNA molecule in biological materials.

Previous studies showed that the recombination frequency could be reduced by the longer extension time during PCR [2], [14], [17]. Analysis of the large number of individual amplicon sequences in this study further demonstrated that extension time had a significant impact on the recombination frequency. The incompletely extended nascent single DNA fragments were considered the main reason for generation recombinants during PCR [2], [12], [14], [27]. At the optimal condition, the *Taq* polymerase can synthesize 1000 bases in less than 10 seconds [45]. However, the manufactures recommend using 1-minute extension time for 1000 bp of the DNA fragment to ensure the complete synthesis of the target templates. The reduced recombination frequencies at longer extension time strongly suggested that the incompletely extended nascent single DNA fragments were still present at such a level that resulted in higher frequencies of recombination at standard recommended extension time. However, the increased extension time could decrease the recombination frequency by reducing the level of the incompletely extended nascent single DNA fragments in the PCR reaction. Thus, longer extension time should be used whenever possible to prevent generation high levels of recombinants during PCR.

When complex microbe quasispecies population [12], [19]–[21] or the immunoglobulin repertoire [22]–[26] is analyzed, the number of genetically distinct templates in the samples is high. The NGS sequence analysis of two different templates showed that the recombination frequency was 14% under the standard PCR condition, but could be significantly reduced under optimized conditions [12]. However, the recombination frequency for less abundant species in a quasispecies population could exceed 70% by NGS analysis [6]. Our NGS analysis results with a mixture of 35 distinct templates showed that as high as 61% of the sequences were recombinants when 3.5×10^6^ templates were amplified with 45 thermal cycles. At the PCR conditions that just generated enough amplicons for subsequent NGS analysis (for example, 30 cycles with 3.5×10^5^ copies or 35 cycles with 3.5×10^4^ copies), the recombination frequency was still 13%. Our results confirmed that the presence of multiple templates could lead to high recombination frequencies and, as in samples with only two templates, more copies of templates and more thermal cycles could result in higher recombination frequencies. Thus, when the quasispecies samples are analyzed by NGS, PCR conditions should be optimized to minimize the number of PCR-derived recombinants.

Recombination among quasispecies templates does not increase the branch length in phylogenetic tree analysis since the mutation rates in the exchanged gene fragments were same as in the parental genomic sequences, but it increases the diversity levels of the genomic population by increasing the number of branches. This was clearly demonstrated in a study in which many additional branches were detected due to the recombinant sequences generated between two homogenous HIV-1 populations by bulk PCR [7]. When the individual viral sequences in the same samples were analyzed by SGS, none of these recombinants were found present. Similar results were also observed with the NGS sequences in our study.

Since various prematurely terminated primer extension fragments are present in the PCR reaction [27] and since heteroduplexes can easily form during a simple hybridization step or a single PCR cycle (nearly 20% of the double strand DNA molecules were heteroduplexes), it is unavoidable to generate recombinants through extension of prematurely terminated primer extension fragments on the different templates when a quasispecies genome population is co-amplified in a single PCR reaction. Although the use of the low template numbers, small thermal cycles and longer extension time can reduce the numbers of recombinants generated during PCR, new technologies are warranted to accurately determine the diversity of a complicated quasispecies gene population.

## Supporting Information

File S1 **Supporting figures and tables.** **Figure S1.** Sequence comparison between templates. Two different HIV-1 strains 89.6 and NL4-3 (A) and two genetic variants 1B7 and 1D1 of HIV-1 strain WEAU (B) were compared to each other. The identical nucleotides are indicated by dash. The nucleotides that were used in the PASS assay to distinguish both templates from each other are indicated by boxes. The positions of each nucleotide used for PASS assay in the PCR amplicon are indicated above the base. The PCR primer sequences are indicated by underline. **Figure S2.** Detection of homoduplexes and heteroduplexes by PASS. The NL4-3 (A) and 89.6 (B) were either amplified individually or as a mixture (C) by PCR. The PCR products were then subjected to PASS analysis. Each polony was annealed to primer 729 and SBE was performed with the Cy3-labeled NL4-3-specific nucleotide (green) and the Cy5-labeled 89.6-specific nucleotide (red). No heteroduplexes were detected in the PCR products amplified from 89.6 or NL4-3 template alone, while a high proportion of the polonies were heteroduplexes (indicated by arrow) in the PCR products amplified from the mixture of 89.6 and NL4-3. **Table S1.** Recombination patterns and frequencies of the PCR amplicons with different thermocycles. **Table S2.** Recombination patterns and frequencies of the PCR amplicons with different input numbers of templates. **Table S3.** Recombination patterns and frequencies of the PCR amplicons with different extension time.
(PDF)
