# Supplementary material for: Extensive Recombination Due to Heteroduplexes Generates Large Amounts of Artificial Gene Fragments during PCR
Source: PLoS One. 2014 Sep 11;9(9):e106658. doi: 10.1371/journal.pone.0106658 (PMC4161356; doi:10.1371/journal.pone.0106658)
Supplement: File S1 — Supporting figures and tables. Figure S1. Sequence comparison between templates. Two different HIV-1 strains 89.6 and NL4-3 (A) and two genetic variants 1B7 and 1D1 of HIV-1 strain WEAU (B) were compared to each other. The identical nucleotides are indicated by dash. The nucleotides that were used in the PASS assay to distinguish both templates from each other are indicated by boxes. The positions of each nucleotide used for PASS assay in the PCR amplicon are indicated above the base. The PCR primer sequences are indicated by underline. Figure S2. Detection of homoduplexes and heteroduplexes by PASS. The NL4-3 (A) and 89.6 (B) were either amplified individually or as a mixture (C) by PCR. The PCR products were then subjected to PASS analysis. Each polony was annealed to primer 729 and SBE was performed with the Cy3-labeled NL4-3-specific nucleotide (green) and the Cy5-labeled 89.6-specific nucleotide (red). No heteroduplexes were detected in the PCR products amplified from 89.6 or NL4-3 template alone, while a high proportion of the polonies were heteroduplexes (indicated by arrow) in the PCR products amplified from the mixture of 89.6 and NL4-3. Table S1. Recombination patterns and frequencies of the PCR amplicons with different thermocycles. Table S2. Recombination patterns and frequencies of the PCR amplicons with different input numbers of templates. Table S3. Recombination patterns and frequencies of the PCR amplicons with different extension time. (PDF) [file pone.0106658.s001.pdf]

A

```
                                             33
89.6    ATAATTGGAAGAAATCTGTTGACTCAGATTGGTTGCACTTTAAATTTTCCCATTAGTCCTATTGAAACTGTACCAGTAAAATTAAAGCCAGGAATGGATGGTCCAAAAG
NL4-3   --------------------------------C--------------------------------G-----------------------------------C-------

                                                                                                         207
89.6    TGAAACAATGGCCATTGACAGAAGAAAAAAATAAAGCATTAGTAGAAATTTGTACAGAAATGGAAAAGGAAGGAAAAATTTCAAAAATTGGGCCTGAGAATCCATACAA
NL4-3   -T-----------------------------------------------------------------------------------------------A-----------

89.6    TACTCCAGTATTTGCCATAAAGAAAAAAGACAGTACTAAATGGAGAAAATTAGTAGATTTCAGAGAACTTAATAAGAGAACCCAAGACTTCTGGGAAGTTCAATTAGGC
NL4-3   ---------------------------------------------------------------------------------T-----T--------------------A

                                                                384
89.6    ATACCACATCCCGCAGGGTTAAAAAAGAAAAAATCAGTAACAGTACTGGATGTGGGTGATGCATATTTTTCAGTTCCCTTAGATGAAGACTTCAGGAAGTACACTGCAT
NL4-3   -----------T------------C-------------------------------C---------------------------A----------------T-------

89.6    TTACCATACCTAGTATAAACAATGAGACACCAGGGATTAGATATCAATACAATGTGCTTCCACAGGGATGGAAAGGATCACCAGCAATATTCCAAAGTAGCATGACAAA
NL4-3   ----------------------------------------------G-----------------------------------------------GT-------------

                                               585                                                         646
89.6    AATCTTAGAGCCTTTTAGAAAACAAAATCCAGACATAGTTATCTATCAATACATGGATGACTTGTATGTAGGATCTGACTTAGAAATAGGGCAGCATAGAGCAAAAATA
NL4-3   ---------------------------------------C--------------------T---------------------------------------A--------

                                                                                  729
89.6    GAGGATCTGAGACAACATCTGTTGAAGTGGGGGTTTACCACACCAGACAAAAAACATCAGAAAGAACCTCCATTTCTTTGGATGGGTTATGAACTCCATCCTGATAAAT
NL4-3   -----A-------------------G------A-----------------------------------------C----------------------------------

89.6    GGACAGTACAGCCTATAGTGCTGCCAGAAAAAGACAGCTGGACTGTCAATGATATACAGAAGTTAGTGGGGAAATTGAATTGGGCAAGTCAGATTTATGCAGGGATT
NL4-3   -------------------------------G--------------------C--------A--------A------------------------------------
```

B

```
1B7     ATAATTGGAAGAAATCTGTTGACTCAGATTGGCTGCACTTTAAATTTTCCCATTAGTCCTATTGAAACTGTACCAGTAAAATTAAAGCCAGGAATGGATGGCCCAAAAG
1D1     -------------------------------------------------------------------------------------------------------------

1B7     TTAAACAATGGCCATTGACAGAAGAGAAAATAAAAGCATTAGTAGAAATTTGTACAGAAATGGAAAAGGAAGGAAAAATTTCAAAAATTGGGCCTGAAAATCCATATAA
1D1     -------------------------------------------------------------------------------------------------------------

                               242
1B7     TACTCCAGTATTTGCCATAAAGAAAAAAGACAGTACTAAATGGAGAAAATTAGTAGATTTCAGAGAACTTAATAAGAGAACTCAAGACTTCTGGGAAGTTCAATTAGGA
1D1     -----------------------G-------------------------------------------------------------------------------------

                          346                                           392
1B7     ATACCACATCCTTCAGGGATAAAAAAGAAAAAATCAGTAACAGTACTGGATGTGGGTGATGCATATTTTTCAGTACCCTTAGATGAAGACTTCAGGAAGTACACTGCAT
1D1     ------------------T---------------------------------------------T--------------------------------------------

1B7     TTACCATACCTAGTATAAACAATGAAACACCAGGGATTAGATATCAGTACAATGTGCTTCCACAGGGATGGAAAGGATCACCAGCAATATTCCAAAGTAGCATGACAAA
1D1     -------------------------------------------------------------------------------------------------------------

                                                             598
1B7     AATATTAGAGCCTTTTAGAAAACAAAATCCAGACATAGTTATCTATCAATACGTGGATGATTTGTATGTAGGATCTGACTTAGAAATAGGGCAGCATAGAACAAAAATA
1D1     ----------------------------------------------------A--------------------------------------------------------

                                             691                            722
1B7     GAGGAGCTGAGACAACATCTGTTGAGGTGGGGATTTACCACACCAGACAAAAAACATCAAAAAGACCATCCATTCCTTTGGATGGGTTATGAACTCCATCCTGATAAAT
1D1     ------------------------------------TA-----------------------------C-----------------------------------------

1B7     GGACAGTACAGCCTATAAAGCTGCCAGAAAAAGAAAGTTGGACTGTCAATGACATACAGAAGTTAGTGGGAAAATTGAATTGGGCAAGTCAGATTTACGCAGGGATT
1D1     -----------------------------------------------------------------------------------------------------------
```

Figure S1. Sequence comparison between templates. Two different HIV-1 strains 89.6 and NL4-3 (A) and two genetic variants 1B7 and 1D1 of HIV-1 strain WEAU (B) were compared to each other. The identical nucleotides are indicated by dash. The nucleotides that were used in the PASS assay to distinguish both templates from each other are indicated by boxes. The positions of each nucleotide used for PASS assay in the PCR amplicon are indicated above the base. The PCR primer sequences are indicated by underline.

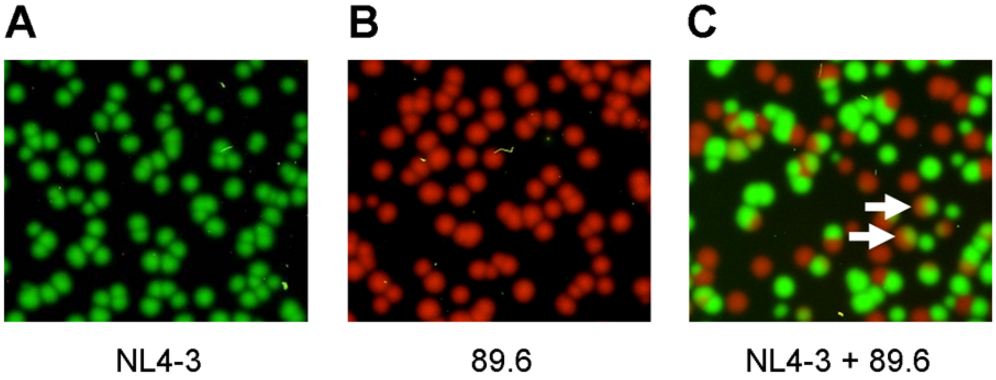

Figure S2. Detection of homoduplexes and heteroduplexes by PASS. The NL4-3 (A) and 89.6 (B) were either amplified individually or as a mixture (C) by PCR. The PCR products were then subjected to PASS analysis. Each polony was annealed to primer 729 and SBE was performed with the Cy3-labeled NL4-3-specific nucleotide (green) and the Cy5-labeled 89.6-specific nucleotide (red). No heteroduplexes were detected in the PCR products amplified from 89.6 or NL4-3 template alone, while a high proportion of the polonies were heteroduplexes (indicated by arrow) in the PCR products amplified from the mixture of 89.6 and NL4-3.

Table S1. Recombination patterns and frequencies of the PCR amplicons with different thermocycles

Pattern						No. of each recombination pattern											
						5 Cycles (697)		10 Cycles (433)		15 Cycles (606)		20 Cycles (420)		25 Cycles (442)		30 Cycles (592)	
33	207	384	585	646	729	No.	%	No.	%	No.	%	No.	%	No.	%	No.	%
+	-	-	-	-	-	--	--	--	--	--	--	--	--	22	4.98	29	4.90
-	+	+	+	+	+	--	--	--	--	--	--	3	0.71	27	6.11	35	5.91
+	+	-	-	-	-	--	--	--	--	--	--	2	0.48	18	4.07	24	4.05
-	-	+	+	+	+	--	--	--	--	--	--	--	--	9	2.04	28	4.73
+	+	+	-	-	-	--	--	--	--	--	--	5	1.19	14	3.17	26	4.39
-	-	-	+	+	+	--	--	--	--	--	--	3	0.71	11	2.49	17	2.87
+	+	+	+	-	-	--	--	--	--	--	--	--	--	1	0.23	8	1.35
-	-	-	-	+	+	1	0.14	--	--	--	--	--	--	5	1.13	9	1.52
+	+	+	+	+	-	--	--	--	--	--	--	--	--	7	1.58	5	0.84
-	-	-	-	-	+	--	--	--	--	1	0.17	1	0.24	4	0.90	6	1.01
-	+	-	-	-	-	--	--	--	--	1	0.17	--	--	1	0.23	8	1.35
+	-	+	+	+	+	--	--	--	--	--	--	--	--	--	--	5	0.84
-	-	+	-	-	-	2	0.29	--	--	1	0.17	--	--	--	--	7	1.18
+	+	-	+	+	+	--	--	--	--	1	0.17	--	--	--	--	5	0.84
-	-	-	+	-	-	1	0.14	--	--	--	--	--	--	--	--	1	0.17
+	+	+	-	+	+	--	--	--	--	--	--	--	--	--	--	1	0.17
-	-	-	-	+	-	--	--	2	0.46	--	--	1	0.24	--	--	--	--
-	+	+	-	-	-	--	--	--	--	--	--	--	--	1	0.23	5	0.84
+	-	-	+	+	+	--	--	--	--	--	--	--	--	2	0.45	3	0.51
-	-	+	+	-	-	1	0.14	--	--	--	--	--	--	2	0.45	1	0.17
-	-	-	+	+	-	--	--	--	--	--	--	--	--	--	--	2	0.34
-	+	+	+	-	-	--	--	--	--	--	--	--	--	--	--	2	0.34
+	-	-	-	+	+	--	--	--	--	--	--	--	--	--	--	2	0.34
-	-	+	+	+	-	--	--	--	--	--	--	--	--	1	0.23	1	0.17
+	+	-	-	-	+	--	--	--	--	--	--	--	--	--	--	2	0.34
-	+	+	+	+	-	--	--	--	--	--	--	--	--	--	--	3	0.51
+	-	-	-	-	+	--	--	--	--	--	--	--	--	1	0.23	--	--
+	-	+	-	-	-	--	--	--	--	--	--	--	--	--	--	2	0.34
-	+	-	+	+	+	--	--	--	--	--	--	--	--	--	--	1	0.17
+	-	-	+	-	-	1	0.14	--	--	1	0.17	--	--	--	--	1	0.17
+	-	-	-	+	-	--	--	--	--	--	--	--	--	--	--	1	0.17
+	-	+	+	-	-	--	--	--	--	--	--	--	--	--	--	1	0.17
+	-	-	+	+	-	--	--	--	--	--	--	--	--	--	--	1	0.17
+	+	-	+	-	-	--	--	--	--	--	--	--	--	--	--	2	0.34
+	+	+	-	+	-	--	--	--	--	--	--	--	--	--	--	1	0.17
-	+	-	-	-	+	--	--	--	--	--	--	--	--	--	--	1	0.17
-	-	-	+	-	+	--	--	--	--	--	--	--	--	--	--	1	0.17
-	-	+	-	+	+	1	0.14	--	--	--	--	--	--	--	--	--	--
-	+	+	+	-	+	1	0.14	--	--	--	--	--	--	--	--	--	--
Total						8	1.15	2	0.46	5	0.83	15	3.57	126	28.51	247	41.72

The plus sign represents the bases specific for 89.6 and the mini sign represents the bases specific for NL4-3. The red vertical bar indicates the purgative recombination breakpoint. The number of PCR amplicons used for analysis are indicated within brackets.

Table S2. Recombination patterns and frequencies of the PCR amplicons with different input numbers of templates

Pattern						No. of each recombination pattern							
						2×10^1copies (487)		2×10^3copies (519)		2×10^5copies (518)		2×10^7copies (592)	
33	207	384	585	646	729	No.	%	No.	%	No.	%	No.	%
+	-	-	-	-	-	--	--	1	0.19	12	2.32	29	4.90
-	+	+	+	+	+	--	--	--	--	17	3.28	35	5.91
+	+	-	-	-	-	--	--	--	--	5	0.97	24	4.05
-	-	+	+	+	+	--	--	--	--	4	0.77	28	4.73
+	+	+	-	-	-	--	--	--	--	1	0.19	26	4.39
-	-	-	+	+	+	--	--	--	--	14	2.70	17	2.87
+	+	+	+	-	-	1	0.21	1	0.19	2	0.39	8	1.35
-	-	-	-	+	+	--	--	--	--	1	0.19	9	1.52
+	+	+	+	+	-	--	--	--	--	2	0.39	5	0.84
-	-	-	-	-	+	--	--	--	--	4	0.77	6	1.01
-	+	-	-	-	-	--	--	--	--	--	--	8	1.35
+	-	+	+	+	+	1	0.21	--	--	--	--	5	0.84
-	-	+	-	-	-	--	--	--	--	1	0.19	7	1.18
+	+	-	+	+	+	--	--	1	0.19	1	0.19	5	0.84
-	-	-	+	-	-	--	--	1	0.19	1	0.19	1	0.17
+	+	+	-	+	+	--	--	--	--	--	--	1	0.17
-	-	-	-	+	-	--	--	--	--	2	0.39	--	--
-	+	+	-	-	-	--	--	--	--	--	--	5	0.84
+	-	-	+	+	+	--	--	--	--	--	--	3	0.51
-	-	+	+	-	-	3	0.62	--	--	--	--	1	0.17
-	-	-	+	+	-	--	--	--	--	--	--	2	0.34
-	+	+	+	-	-	--	--	--	--	--	--	2	0.34
+	-	-	-	+	+	--	--	--	--	--	--	2	0.34
-	-	+	+	+	-	--	--	--	--	--	--	1	0.17
+	+	-	-	-	+	--	--	--	--	--	--	2	0.34
-	+	+	+	+	-	--	--	--	--	1	0.19	3	0.51
+	-	+	-	-	-	--	--	--	--	--	--	2	0.34
-	+	-	+	+	+	--	--	--	--	--	--	1	0.17
+	-	-	+	-	-	--	--	--	--	--	--	1	0.17
+	-	-	-	+	-	--	--	--	--	--	--	1	0.17
+	-	+	+	-	-	--	--	--	--	--	--	1	0.17
+	-	-	+	+	-	--	--	--	--	--	--	1	0.17
+	+	-	+	-	-	--	--	--	--	--	--	2	0.34
+	+	+	-	+	-	--	--	--	--	--	--	1	0.17
-	-	-	+	-	+	--	--	--	--	--	--	1	0.17
-	+	-	-	-	+	--	--	--	--	--	--	1	0.17
Total						5	1.03	4	0.77	68	13.13	247	41.72

The plus sign represents the bases specific for 89.6 and the mini sign represents the bases specific for NL4-3. The red vertical bar indicates the purgative recombination breakpoint. The number of PCR amplicons used for analysis are indicated within brackets.

Table S3. Recombination patterns and frequencies of the PCR amplicons with different extension time

Pattern						No. of each recombination patterin in different minutes (min)							
						1 min (348)		2 min (401)		4 min (405)		8 min (438)	
33	207	384	585	646	729	No.	%	No.	%	No.	%	No.	%
+	-	-	-	-	-	28	8.05	8	2.00	13	3.21	5	1.14
-	+	+	+	+	+	15	4.31	10	2.49	11	2.72	2	0.46
+	+	-	-	-	-	18	5.17	13	3.24	9	2.22	--	--
-	-	+	+	+	+	16	4.60	11	2.74	4	0.99	1	0.23
+	+	+	-	-	-	16	4.60	3	0.75	3	0.74	3	0.68
-	-	-	+	+	+	10	2.87	11	2.74	2	0.49	1	0.23
+	+	+	+	-	-	4	1.15	3	0.75	1	0.25	--	--
-	-	-	-	+	+	1	0.29	5	1.25	1	0.25	--	--
+	+	+	+	+	-	7	2.01	2	0.50	3	0.74	2	0.46
-	-	-	-	-	+	4	1.15	5	1.25	--	--	5	1.14
-	+	-	-	-	-	2	0.57	2	0.50	--	--	--	--
+	-	+	+	+	+	--	--	2	0.50	--	--	--	--
+	+	-	+	+	+	1	0.29	--	--	1	0.25	--	--
+	+	+	-	+	+	2	0.57	--	--	--	--	--	--
+	+	+	+	-	+	1	0.29	--	--	--	--	--	--
-	-	-	-	+	-	--	--	1	0.25	--	--	--	--
-	+	+	-	-	-	--	--	4	1.00	--	--	--	--
+	-	-	+	+	+	1	0.29	1	0.25	--	--	--	--
+	-	-	-	+	+	--	--	--	--	--	--	1	0.23
-	+	+	+	-	-	1	0.29	--	--	1	0.25	--	--
+	-	-	-	-	+	--	--	--	--	1	0.25	--	--
-	+	+	+	+	-	1	0.29	--	--	--	--	--	--
+	-	+	+	+	-	3	0.86	--	--	--	--	--	--
-	-	-	+	-	+	--	--	--	--	1	0.25	--	--
Total						131	37.64	81	20.20	51	12.59	20	4.57

The plus sign represents the bases specific for 89.6 and the mini sign represents the bases specific for NL4-3. The red vertical bar indicates the purgative recombination breakpoint. The number of PCR amplicons used for analysis are indicated within brackets.
